# Development and Validation of Videogame Addiction Scale for Children (VASC)

**DOI:** 10.1007/s11469-017-9766-7

**Published:** 2017-05-15

**Authors:** Eyüp Yılmaz, Mark D. Griffiths, Adnan Kan

**Affiliations:** 1 Psychology Department, Nottingham Trent University, Nottingham, UK; 2 International Gaming Research Unit, Psychology Department, Nottingham Trent University, Nottingham, UK; 3 Division of Psychology, Gazi University, Ankara, Turkey

**Keywords:** Gaming, Videogames, Videogame addiction, Gaming addiction screening, Youth gaming

## Abstract

The aim of the present study was to develop a valid and reliable Videogame Addiction Scale for Children (VASC). The data were derived from 780 children who completed the Videogame Addiction Scale (405 girls and 375 boys; 48.1% ranging in age from 9 to 12 years). The sample was randomly split into two different sub-samples (sample 1, *n* = 400; sample 2, *n* = 380). Sample 1 was used to perform exploratory factor analysis (EFA) to define the factorial structure of VASC. As a result of EFA, a four-factor structure comprising 21 items was obtained and explained 55% of the total variance (the four factors being “self-control,” “reward/reinforcement,” “problems,” and “involvement”). The internal consistency reliability of VASC has found 0.89. Confirmatory factor analysis (CFA) was performed to confirm the factorial structure obtained by EFA in the remaining half of sample (*n* = 390). The obtained fit indices from the CFA confirmed the structure of the EFA. The 21-item VASC has good psychometric properties that can be used among Turkish schoolchildren populations.

With the rapid development of technology and the widespread use of Internet, new forms of leisure activity (online videogames, social networking, etc.) have emerged and are engaged in by many individuals including children and adolescents. The videogame industry has become one of the largest industries in terms of both money and audience reached, and for a small minority, the playing of videogames has been reported as being potentially problematic and/or addictive (Gentile 2011; Gentile and Wash 2002).

The latest generation of videogames possesses more stimulating visual and auditory effects, in addition to rapid event frequencies that encourage continuous use (Ng et al. 2005). This can lead to individuals spending excessive amounts of time on videogames. Motivations and expectancies can also play a role in excessive gaming (Haagsma et al. 2013). This has led to claims that overusing videogames can lead to a behavioral addiction (Griffiths 2005a). Research has demonstrated that male adolescents are more vulnerable to developing problematic gaming habits and more likely to show signs of pathological gaming than older age groups (Griffiths and Wood 2000; Haagsma et al. 2012).

Many researchers use various terms for videogame addiction including (but not limited to) excessive gaming, problematic gaming use, Internet gaming disorder, compulsive videogame playing, pathological gaming, videogame addiction, digital game addiction, online gaming addiction, and Internet gaming addiction (American Psychiatric Association 2013; Faust and Faust 2015; Gunuc 2015; Huanhuan and Su 2013; Kim and Kim 2010; Kim et al. 2008; Kuss and Griffiths 2012; Lemmens et al. 2011). Irrespective of what it is called, there are similarities in symptoms between addictive behaviors and problematic gaming. Problematic gamers display behavioral tendencies found in more traditional addiction disorders (Van-Holst et al. 2012).

Griffiths (1995) has also claimed that there are two sub-types of addiction referred to as primary and secondary addictions. In primary addictions, the person is addicted to the activity itself and loves engaging the activity. In secondary addiction, the individual engages in the behavior as a way of dealing with other underlying problems. Griffiths (2005a) claims that primary addictions are more resistant to treatment because the person genuinely loves that activity and would not want to willingly give it up whereas secondary addictions are easier to treat because the excessive activity is symptomatic of underlying problems. If the underlying problem is addressed therapeutically, the addictive behavior should diminish. While online gaming addiction was not recognized as a pathological disorder in the fourth edition of the *Diagnostic and Statistical Manual of Mental Disorders* (American Psychiatric Association [APA] 1994), it features in the in the appendix of the DSM-5 as 'Internet Gaming Disorder.'

The concept of gaming addiction has different core features depending upon the researchers. These include being unable to control the action and continuing the behavior in spite of its negative results (Henderson 2001), an excessive and compulsive use of videogames that results in social and/or emotional problems and that despite these problems, and the gamer being unable to control this excessive use (Lemmens et al. 2009). The APA (2013) define IGD as: “A pattern of excessive and prolonged Internet gaming that results in a cluster of cognitive and behavioral symptoms, including progressive loss of control over gaming, tolerance, and withdrawal symptoms, analogous to the symptoms of substance use disorders” (APA 2013, pp.796).

Videogame playing has a number of factors that make it an attractive and rewarding activity such as social recognition from other players (King and Delfabbro 2009), deriving satisfaction from being part of group and playing for relaxation (Yee 2006), mood regulation, sensation seeking, relieving feelings of boredom and loneliness (Lee and LaRose 2007; Zuckerman 1994), providing escape from daily life problems (Wan and Chiou 2006a, b), and the developing of intimate relationships with others in an imaginary game world that is easier than face-to-face communication (Huanhuan and Su 2013).

Compared to older video-arcade games and non-continuous forms of gaming, newer computer games and online Internet activities have greater salient effects, stimulating visual and auditory effects and rapid event frequency, which encourage continuous use (Brown and Robertson 1993; Griffiths and Hunt 1998; Ng and Wiemer-Hastings 2005). This is worrisome because such gaming activities and interactive online media are becoming increasingly popular among youth and are proposed to be more addictive than video-arcade games and non-continuous forms of gambling (Chou and Hsiao 2000; Griffiths et al. 2004; Whang et al. 2003).

The playing of videogames has been discussed in relation to whether the activity is beneficial or harmful for individuals, especially for children and adolescents. Over the past few decades, a growing body of studies has attempted to contribute to the debate. However, it is clear that gaming can have both positive and negative effects depending upon the content and context of game playing. While gamers may spend a lot of time playing videogames, they may also improve their visual attention skills simultaneously (Gentile 2011). There are a considerable number of studies that have found significant associations between excessive online game playing and high levels of depression, anxiety, aggression, trait anxiety, neuroticism, loss of appetite, sleep disturbance, and neglect of physical activity (e.g., Anderson and Murphy 2003; Charlton 2002; Chumbley and Griffiths 2006; Wallenius et al. 2007). Research has also found associations between excessive and compulsive videogame use with poor psychosocial well-being (Lemmens et al. 2011), less satisfaction with daily life (Wang et al. 2008), poor scholastic performance (Gentile et al. 2004; Skoric et al. 2009; Rehbein et al. 2010), and aggression and narcissism (Kim et al. 2008). However, there are many studies that have found positive aspects of videogame playing including high intrinsic motivation (Wan and Chiou 2007), entertainment value (Lim and Lee 2009; Thomas and Martin 2010), educational, social, and/or therapeutic benefits (Griffiths 2002, 2005b; Griffiths 2010a, b), skills enhancement (Gee 2007; Dickey 2011), simulation opportunities to explore environments without risk (Aldrich 2005), and knowledge promotion of computer memory concepts (Papastergiou 2009).

Videogame playing has become one of children’s favorite leisure activities. They appear to prefer videogames over television due to the greater control opportunities and active involvement (Greenfield 1984). In the USA, children aged between 2 and 17 years play videogames for an average of 7 h a week (Gentile and Walsh 2002). In another study, children aged between 9 and 12 years from 12 different countries (Australia, Brazil, Canada, China, Colombia, Finland, India, Kenya, Portugal, South Africa, UK, and USA) spent an average of 8.6 h engaged in sedentary behavior including videogame playing (LeBlanc et al. 2015). According to the China Internet Network Information Center (CNNIC 2016), children/adolescents who are aged from from 10 to 19 years constituted 20.1% of all Internet users in China. Christakis et al. (2004) reported that 5-year-old children spent approximately 4 h a week playing videogames. Studies on Turkish children have been shown that children aged 3 to 18 years prefer to play videogames using their own devices (Arnas 2005; Deveci et al. 2007).

There have been many scales developed to assess problematic videogame play. In a systematic review, King et al. (2013) identified 18 different screening instruments including instruments based on gambling or Internet addictions. Some of the most used screens include the Game Addiction Scale (GAS) developed by Lemmens et al. (2009) for adolescents. The scale was developed with data derived from 644 Dutch adolescents aged 12 to 18 years (mean age 14.8 years; SD = 1.64). The GAS also has been adapted for Brazilian adolescents (Lemos et al. 2016), for Turkish adolescents and adults (Baysak et al. 2016), and for French adolescents (Gaetan et al. 2014). A more recent Internet Gaming Disorder Scale Short Form (IGDS-SF9) was developed by Pontes and Griffıths (2015). The 7-Item Game Addiction Scale was developed by Khazaal et al. (2016) for French and German speaking adults. Wan and Chiou (2006a, b) developed the Online Game Addiction Scale for Taiwan Adolescents (OAST) for those older than 12 years old. The Problem Videogame Playing (PVP) Scale was developed by Salguero and Moran (2002) for those 13 years old and over. Furthermore, the Videogame Addiction Test (Van Rooij et al. 2012) and Videogame Dependency Scale (Rehbein et al. 2010) were developed for 13-year-old adolescents or over age groups.

Considering the current videogame addiction screens (all of them for those aged over 12 years), we believe that a new videogame addiction scale is needed for young children. There is also a Computer Game Addiction Scale that was developed by Horzum et al. (2008) among Turkish children sample. When the scale items are examined, it can be seen that all of them refer to “playing on a computer”. In this case, the children who play videogames but not via their computer (i.e., mobile phone, tablet, *Xbox* console, *PlayStation* console etc.) cannot endorse these items. In addition, unlike this study, we aimed to perform confirmatory factor analysis (CFA) to confirm the factor structure obtained via exploratory factor analysis (EFA) and so have an assessment instrument in which validity is tested. We also aimed to compare the factor structure with a larger sample as an another reason to develop a new videogame addiction scale for children. Consequently, the main purpose of this study was to develop a valid and reliable Videogame Addiction Scale for Children (VASC).

## Method

### Participants

The data for scale development were derived from 780 children (405 girls [51.9%] and 375 boys [48.1%] ranging in age from 9 to 12 years; mean age = 10.36 years, *SD* = 0.49) who participated in the study over a 3-month period (December 2015 to February 2016). Detailed descriptive statistics of the participants are provided in Table 1.

**Table 1:** Detailed descriptive statistics about participants (*N* = 780)

Gender	Class level						Total	
	Fourth grade (9–10)		Fifth grade (10–11)		Sixth grade (11–12)			
	*N*	%	*n*	%	*n*	%	*n*	%
Girl	140	51.7	141	52.8	124	51.2	405	51.9
Boy	131	48.3	126	47.2	118	48.8	375	48.1
Total	271	100	267	100	242	100	780	100

### Procedure

During the process of developing of the draft version of the scale, the related literature and similar scale development studies were reviewed (e.g., DSM-5 2013; Geaten et al. 2014; Khazaal et al. 2016; Mak et al. 2014; Lemmens et al. 2009; Lemos et al. 2016; Pontes et al. Griffiths 2015; Pontes et al. 2016; Spekman et al. 2013). The draft scale comprised 31 items. To enable the face and language validity of scale, five Turkish specialists were consulted (an academic from the Division of Assessment and Evaluation in Education, two academic psychologists, a primary school teaching academic, and two classroom teachers). Scale items were also read by nine children (all 9 years old). Items that were not fully understood by the children were replaced until all the items were understood by all nine children.

After ethical approval was granted from the research team’s University Ethical Committee, permission to carry out the study was approved by the Turkish Provincial National Education Directorate. Prior to the study, children, the school Principal, and relevant teachers were informed about the purpose of the study. A consent form sent to parents also asked permission for their child to participate in the study. Children assured that their responses would remain confidential and anonymous, and only viewed by the research team (and not shown to their teachers, parents, or friends). A total of 850 children began completing the questions with 780 children completing all of them. Analysis was only carried out on completed questionnaires. Children completed the questionnaire in approximately 25 minutes.

### Assessment Materials

#### Videogame Addiction Scale for Children

The draft form of Videogame Addiction Scale for Children (VASC) comprised 21 items (see Appendix 1 for the Turkish version that was used in the present study; 31 items before the exploratory factor analysis). All items were scored on a five-point scale by the children (“never” = 1, “rarely” = 2, “sometimes” = 3, “often” = 4, “very often” = 5). Total scores are obtained by summing the children’s response scores and total scores range from 21 to 105. The present authors’ recommendation is that a score above 90 indicates a possible addiction to videogames. It is also stressed that this is not a diagnostic tool but only an indicator that a child might have an addiction to videogames. Only an in-depth clinical assessment could provide such a diagnosis.

#### Socio-Demographics

The survey included questions relating to gender, age, socio-economic situation, and whether children had access to technological devices that allowed them to play videogames (personal computer, laptop, tablet, mobile phone, etc.). While the majority of participants (59.3%; *n* = 463) had at least one technological device of their own, one-third of the participants (32.9%; *n* = 257) had to share their technological devices with their siblings, and a small number of participants (7.8%; *n* = 60) did not have their own technological device. One-fifth of participants (21.1%; *n* = 165) had a low socio-economic status, almost a half of participants (46.2%; *n* = 360) had medium socio-economic status, and one-third of participants (32.7%; *n* = 255) had high socio-economic status. Furthermore, boys’ VASC scores (*N* = 341, *M* = 57.95, *SD* = 16.49) were significantly higher than the girls’ VASC scores (*N* = 379, *M* = 43.7, *SD* = 12.11) *t* = 43.7, *p* < 0.01.

### Statistical Analysis

To test the construct validity of the VASC, an exploratory factor analysis (EFA) was carried out. This was done to test the quality of items, item discrimination index, item difficulty index, item-total correlation analysis, and Cronbach’s alpha reliability analysis. All analysis was carried via IBM SPSS 21. To be evaluated as a robust scale, the eigen value had to be at least 1, the factor loading values of the scale items had to be at least .40, and the load values of items which loaded on more than one factor had to be more than .20 between the load values. To confirm the obtained factor structure of the EFA, confirmatory factor analysis (CFA) was performed using the LISREL 8.80 statistical program. All statistical tests were performed using a significance level of *p* < 0.05.

Before testing the factor structure of the Videogame Addiction Scale for Children (VASC), the whole sample was randomly split into the two groups (Sample 1, *n* = 400, *M* = 2.35, *SD* = 0.75; Sample 2, *n* = 380, *M* = 2.43, *SD* = 0.78). The data of one of these groups was used to determine the dimensional structure of the VASC and the other one was used to confirm the obtained dimensional structure.

## Results

### Exploratory Factor Analysis

As a first step, Kaiser-Meyer-Olkin (KMO) coefficient and Bartlett’s test of Sphericity were performed in order to find out whether the VASC is appropriate for principal component analysis. The threshold for the KMO value should be .60 and the data set for KMO < .60 cannot be factored (Field 2000; Pallant and Manual 2001). As a result of analysis, it was found that the dataset was suitable for dimensional structure analysis (KMO = .91, χ^2^ = 2770.019; *p* < 0.01). EFA was performed via oblimin with Kaiser normalization rotation method to obtain the dimensional structure. Tabachnick and Fiddell (2007) advise looking at the correlations among the factors when trying to decide between orthogonal and oblique rotation (i.e., varimax, direct oblimin, or promax). If the correlations exceed 0.32, then there is 10% (or more) overlap in variance among factors and that means enough variance to warrant oblique rotation. Having found correlations over 0.32 between factors (see Table 2), oblimin with Kaiser normalization rotation was performed (Table 3).

**Table 2:** Correlations among the subscales of the Videogame Addiction Scale for Children

	Self-control	Reward/reinforcement	Problems	Involvement
Self-control	1	.534	.390	.625
Reward/reinforcement		1	.120	.518
Problems			1	.277
Involvement				1

*p* < 0.01

**Table 3:** Validity and reliability coefficients of the Videogame Addiction Scale for Children

Factor name	Item number	Item	Factor load	Cronbach’s alpha (sub-scale)	Cronbach’s alpha (scale)
Self-control	27	I cannot resist playing videogames even if it negatively affects my life	*.718*	*0.84*	*0.89*
	30	Even if I control the amount of time I spend playing videogames, after a while I continue to play again uncontrollably	*.718*		
	26	I feel that whatever I do, I am not able to control the time I spend playing videogames	*.656*		
	20	I cannot stop playing videogames even if I think I have spent so much time playing them	*.651*		
	18	I am not interested in anything else while playing videogames	*.644*		
	25	Although I want to reduce amount of time I spend playing videogames, I fail every time	*.640*		
	14	I forget my problems while playing videogames	*.483*		
Reward/reinforcement	3	In videogames, defeating my enemies/leaping up a level gives me pleasure	*.776*	*0.83*	
	15	In videogames, defeating my enemies/leaping up a level makes me feel stronger than my enemies.	*.706*		
	8	I think playing videogames is very enjoyable activity	*.700*		
	17	In videogames, defeating my enemies/leaping up a level increases my self-esteem	*.693*		
	13	I do not feel bored when I play videogames	*.660*		
	29	I feel happy when I play videogames	*.642*		
Problems	9	Playing videogames prevents me from fulfilling my responsibilities	*.862*	*0.75*	
	5	Playing videogames prevents me from eating regular meals	*.750*		
	2	The games I play prevent me from spending time with my family	*.746*		
	10	I have sleeping problems due to playing videogames	*.490*		
Involvement	23	I always talk about videogames with my friends	*.683*	*0.73*	
	28	I make friends via online videogames	*.673*		
	22	I see my videogames/game characters in my dreams	*.624*		
	16	I act like videogame characters in my daily life activities	*.513*		

Ten items that loaded on more than one factor and had less than .20 factor load difference between factor loads or had less than .40 factor load were removed from scale (i.e., items 1, 4, 6, 7, 11, 12, 19, 21, 24, and 31) including some items that may be indicative of videogame addiction such as not getting enough sleep and missing meals. A four-factor structure (“self-control,” “reward/reinforcement,” “problems,” and “involvement”) comprising 21 items remained that had an eigenvalue greater than 1. Furthermore, Cronbach’s alpha coefficient, item-total, and inter-item correlations were computed to examine the internal consistency of the VASC. As a result of reliability analysis, the internal consistency reliability (Cronbach’s alpha) of the scale and sub-scales were satisfactory (VASC = 0.89, self-control = 0.84, reward/reinforcement = 0.83, problems = 0.75, involvement = 0.73). The results indicated that item-total correlations for 21 items ranged from 0.483 to 0.862. The four factors explained the 55.7% of total variance (self-control = 3.686, 17.550; reward/reinforcement = 3.449, 16.425; problems = 2.416, 11.506; involvement = 2.146, 10.218).

### Confirmatory Factor Analysis

**Fig. 1:**
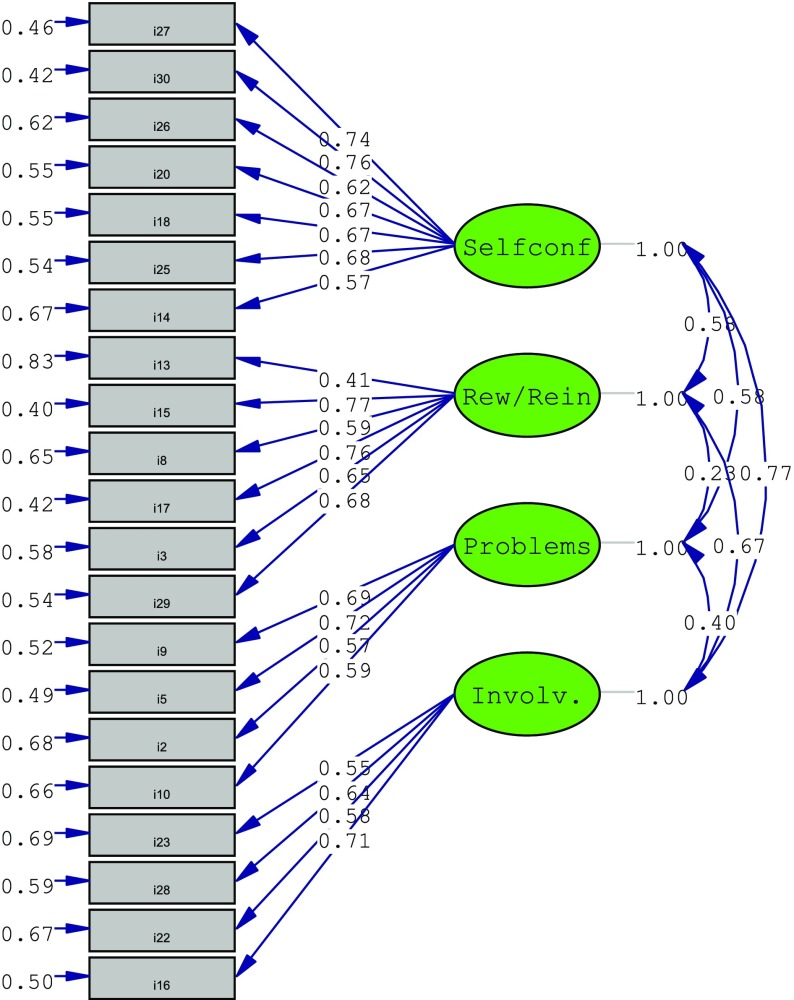
Standardized scores of four-factor structure of the Videogame Addiction Scale for Children

Confirmatory factor analysis was performed on the 21 items of the VASC using Sample 2 (*n* = 380; *M* = 2.43, *SD* = 0.78) in order to confirm the four-factor structure (self-control, reward/reinforcement, problems, and involvement) using the Lisrel 8.8 statistical program. The standardized path diagram scores of the four-factor structure are shown in Fig. 1. The four-factor structure resulted in an acceptable model fit and confirmed the four-factor structure obtained via EFA *X* ^*2*^(183, *N* = 380) = 535.01, *p* < .001. Chi-square (*X* ^*2*^) is a traditional measure that used to assess the fit of the model and to determine the magnitude of the discrepancy between covariance matrices (Hu and Bentler 1999).

The CFA also provided the following results: *X* ^*2*^ = 2.9 and *p* > .05, root mean square error of approximation (RMSEA) = .05, goodness of fit index (GFI) = .91, adjusted goodness of fit index (AGFI) = .90, standardized root mean square residual (SRMR) = .08, normed fit index (NFI) = .93, non-normed fit index (NNFI) = .96, and comparative fit index (CFI) = .96. Below 0.08 of RMSEA fit indices indicates good harmony (MacCallum et al. 1996); between .90 and .95 of CFI and AGFI fit in indexes are considered as acceptable harmony, and over .95 on these indexes is considered perfect harmony (Hooper et al. 2008; Miles and Shevlin 2007); up to 0.08 of SRMR can be considered acceptable harmony (Byrne 1998); between .90 and .95 of NFI and NNFI is considered acceptable harmony; and over .95 on these fit indexes are considered perfect harmony (Hu and Bentler 1999). All of these results indicate that four-factor model has an excellent fit and confirms the dimensional structure obtained via EFA.

## Discussion

The main purpose of the present study was to develop a valid and reliable screening instrument to assess videogame addiction among pre-adolescent children (i.e., the Videogame Addiction Scale for Children [VASC]). Based on review of the literature, 31 items which have been associated with problematic videogame play were developed. To assess the psychometric characteristics of the VASC, data from the whole sample was randomly split into the two groups (sample 1 = 400 participants; sample 2 = 380 participants). While Sample 1 was used to determine the dimensional structure of VASC using EFA, Sample 2 was used to confirm the dimensional structured which is obtained using CFA. As a result of the EFA, a four-factor structure [self-control (7 items), reward/reinforcement (6 items), problems (4 items), and involvement (4 items)] emerged comprising 21 items. Confirmatory factor analysis provided evidence of the validity of four-factor structure.

When similar problematic videogame playing scales are examined, some researchers have items based on core addiction components (i.e., salience, tolerance, mood-modification, withdrawal, relapse, conflict, and problems) proposed by Griffiths (2005a) such as the Game Addiction Scale (GAS; Lemmens et al. 2009), the 20-item Internet Gaming Disorder Test (IGD-20; Pontes et al. 2014), Problem Videogame Playing (PVP) Scale (Salguero and Moran 2002), Videogame Addiction Test (Van Rooij et al. 2012), and Young Internet Addiction Scale (YIAS; Young 1996). However, some of scale studies have reported a different dimensional structure such as when validating the Game Addiction Scale Among French and German speaking adults (Khazaal et al. 2016) and the Online Game Addiction Index (OGAI; Zhou and Li 2009; Demetrovics et al. 2012). In the present study, a different dimensional structure (i.e., four-factor structure) was found because the scale items were produced independently of known addiction components. The Cronbach’s alpha reliability coefficient was found to be good and Sample 2 confirmed the four-factor structure (i.e., self-control, reward/reinforcement, problems, and involvement).

The result of boys’ scores on the VASC was significantly higher than girls’ scores. This is similar to the findings of Huanhuan and Su (2013) who found significantly higher levels of Internet addiction and gaming addiction among boys compared to girls. Brunborg et al. (2013) reported the prevalence of game addiction to be 6.5% for boys and 2.2% for girls, with boys playing a mean average of 15 h a week compared to 5 h a week for girls. Similar findings have also been found in other studies (e.g., Lemmens et al. 2011; Zorbaz et al. 2015).

The study is not without its limitations. The sample was self-selecting and utilized data collected from Turkish children. Consequently, the findings are not representative of Turkish children and may not be generalizable to other countries. For validation studies, representativeness is not a major issue, and arguably, over-sampling excessive videogame players is more important. However, the present authors were unable to find a way of over-sampling such a group in the way permission was sought to recruit participants, so this is another limitation that needs to be taken into account. All the data were self-report and there suffer from well-known biases including social desirability biases (although the age of sample might be a mitigating factor in this particular instance) and memory recall biases. The initial dataset included responses from 840 children, but data from 60 children (7% of the initial sample) were discarded due to incomplete responses or recording the same response to every question. It can only be speculated as to why these 60 children did not complete the questionnaire properly (e.g., boredom, simply not wanting to participate even though informed consent had been given by their parents) or whether the omission had any impact on the results found (although the present authors suspect not). Also, there were no data collected that assessed the criterion validity of the VASC (e.g., time spent playing videogames, corroborating evidence from teachers and/or parents, etc.). Such data would be useful to collect in future studies. Furthermore, the scale was designed to be used in epidemiological studies rather than in a clinical context, but given the nature of the instrument, in-depth exploration using items in the scale could be used in a clinical context to get a detailed picture of the psychosocial impact of videogame playing in children’s lives.

Given that the scale study was developed via Turkish children sample, the VASC needs validating in other languages and cultures to further test the four-factor structure, validity, and reliability of VASC. It also needs further testing among different cohorts of Turkish schoolchildren as the sample in the present study was not necessarily representative. Based on the sample that was tested, it is concluded that the 21-item VASC has good psychometric properties and an established four-factor model confirming the structure with an excellent fit.

## Appendix 1 Turkish version of the Videogame Addiction Scale for Children

ÇOCUKLAR İÇİN VİDEO OYUNLARI BAĞIMLILIĞI ÖLÇEĞİ.

Aşağıdaki sorularda size en uygun olan bölüme (X) işareti koyunuz.

**Table:**

Sıra No	Madde	Hiç	Nadiren	Bazen	Genellikle	Her Zaman
27	Günlük hayatımda birçok olumsuzluğa neden olsa bile oyun oynamaktan kendimi alamam.					
30	Oyunlara harcadığım süreyi kontrol altına alsam bile birkaç gün sonra tekrar kontrolsüz bir şekilde oynamaya devam ederim.					
26	Oyunlara harcadığım süreyi ne yaparsam yapayım kontrol edemeyeceğimi düşünürüm.					
20	Çok uzun süre oyun oynadığımı düşünsem bile oynamayı bırakamıyorum.					
18	Oyun oynarken çevremdeki başka hiçbir şey ilgimi çekmez					
25	Oyunlara harcadığım süreyi azaltmak istememe rağmen her defasında başarısız olurum.					
14	Oyun oynarken hayatımdaki sorunları unuturum.					
3	Oyunda rakiplerimi yenmek, seviye atlamak ya da en fazla puanı toplamak bana zevk verir.					
15	Oyunda rakiplerimi yenmek ya da üst seviyeye çıkmak onlardan daha güçlü olduğum hissini verir.					
8	Oyun oynamanın eğlenceli bir etkinlik olduğunu düşünürüm.					
17	Oyunda düşmanlarımı yenmek/seviye atlamak kendime olan güvenimi artırır.					
13	Oyun oynarken çok sıkılırım.					
29	Oyun oynarken kendimi mutlu hissederim.					
9	Oyun oynamak sorumluluklarımı yerine getirmemi engeller.					
5	Oyun oynamak düzenli bir şekilde yemek yememi engeller.					
2	Oynadığım oyunlar ailemle vakit geçirmeme engel olur.					
10	Oyun oynadığım için uyku problemleri yaşıyorum.					
23	Arkadaşlarımla, oynadığım oyunlar hakkında konuşurum.					
28	Oyunlar sayesinde internetten arkadaşlıklar edinirim.					
22	Oynadığım oyunlar ya da oyun karakterleri rüyalarıma bile girer.					
16	Günlük yaşamdaki aktivitelerimde kendimi oyun içerisinde gibi hissederim.					
